# Predictive factors of success at the French National Ranking Examination (NRE): a retrospective study of the student performance from a French medical school

**DOI:** 10.1186/s12909-019-1903-5

**Published:** 2019-12-21

**Authors:** Martin Lhuaire, Moustapha Dramé, Mikael Hivelin, Thomas Levasseur, Quentin Maestraggi, Vincent Hunsinger, Peter Abrahams, Laurent Lantieri, Daniele Sommacale

**Affiliations:** 1 Department of General and Digestive Surgery, Hôpital Robert Debré, Centre Hospitalier Universitaire de Reims, Université de Reims Champagne-Ardenne, Avenue du Général Koenig, 51092 Reims, France; 2 Department of Plastic, Reconstructive and Aesthetic Surgery, Hôpital Européen Georges Pompidou, Assistance Publique des Hôpitaux de Paris, Université Paris Descartes, Paris, France; 3 Department of Research, Innovation and Biostatistics, Hôpital Pierre Zodba-Quitman, Centre Hospitalier Universitaire de Martinique, Université des Antilles, Fort de France, Martinique France; 4 Department of Cardiology, Hôpital Robert Debré, Centre Hospitalier Universitaire de Reims, Université de Reims Champagne-Ardenne, Reims, France; 5 Department of Intensive Care Unit, Hôpital de Hautepierre, Université de Strasbourg, Strasbourg, France; 6 Institute of Anatomy and Clinical Education, Warwick Medical School, University of Warwick, Coventry, CV4 7AL UK

**Keywords:** National ranking examination, Prognostic study, Predictive factors of success, Medical pedagogy

## Abstract

**Background:**

The national ranking examination (NRE) marks the end of the second cycle (6th university year) of French medical studies and ranks students allowing them to choose their specialty and city of residency. We studied the potential predictive factors of success at the 2015 NRE by students attending a French School of Medicine.

**Methods:**

From March 2016 to March 2017, a retrospective study of factors associated with the 2015 NRE success was conducted and enrolled 242 students who attended their sixth year at the school of medicine of Reims. Demographic and academic data collected by a home-made survey was studied using univariate and then multivariate analysis by generalized linear regression with a threshold of *p* <  0.05 deemed significant.

**Results:**

The factors independently associated with a better ranking at the NRE were the motivation for the preparation of the NRE (gain of 3327 ± 527 places, *p* <  0.0001); to have participated in the NRE white test organized by *la Revue du Praticien* in November 2014 (gain of 869 ± 426 places, *p* <  0.04), to have participated in the NRE white test organized by *la conférence Hippocrate* in March 2015 (+ 613 places ±297, p <  0.04). The factors independently associated with poor NRE ranking were repeating the first year (loss of 1410 places ±286, p <  0.0001), repeating a year during university course (loss of 1092 places ±385, *p* <  0.005), attendance of hospital internships in 6th year (loss of 706 places ±298, *p* <  0.02).

**Conclusions:**

The student motivation and their white tests completion were significantly associated with success at the NRE. Conversely, repeating a university year during their course and attendance of 6th year hospital internships were associated with a lower ranking.

## Background

The national ranking examination (NRE) marks the end of the second cycle of french medical studies. All french medical students must participate in the examination at the end of the 6th year of their university medical curriculum before beginning their residency (third cycle of french medical studies). Following this very selective examination (ranking of 8908 candidates in 2015), students are ranked according to their performance. The rank obtained is decisive for the student’s professional career, since it allows them to choose the specialty and the city in which they will complete the residency (M.D degree) [1, 2]. The main objective of the NRE is to rank medical students, but it also allows ranking right across all 35 french medical schools on the basis of their students performances at this examination [3, 4]. Since 2010, a yearly statistical analysis of the results of the NRE gives the ranking of French medical schools, which remains substantially identical from year to year [4–9]. In this context, it seems obvious that a given student, regardless of their potential for personal success, does not have the same probability of success at NRE dependent on their medical school of attendance. There are indeed disparities in terms of quality of education and therefore a real inequality of opportunity depending on the geographical location of medical students in France. Indeed, Karila et al., had demonstrated in 2011 that attending medical school in *Île-de-France* (Paris) was independently and significantly associated with a better ranking at the NRE [10]. Rabineau and Dhainaut in 2010 reinforces this unequal picture by demonstrating that there is a triple correlation between the percentage of students ranked in the top 1000 at the NRE, the research activity of university hospital practitioners and the ratio of university hospital practitioners relative to the number of students from different medical schools [3]. Every year amongst, the medical school of Reims is invariably ranked the lowest in France in terms of success of its students at the NRE [4–9]. In this sense, it seemed interesting to determine whether the predictive factors for NRE success previously described in the literature were influenced by the student’s school of medicine of attendance. Lastly, it seemed only sensible to identify objectively the existence of predictive factors of success and to analyze their impact on an individual level.

The objective of this study was to determine the predictive factors of success at the 2015 NRE of the 6th year medical students attending the medical school of Reims.

## Methods

### Study design

Between March 2016 and March 2017, a retrospective study of factors associated with the 2015 NRE success was carried out and enrolled 242 students who attended their sixth year at the Reims medical school.

### Data management

Demographic, academic and results data of the white test examinations organized by the private NRE training conferences (*Conférence Hermès, SASU, 11 rue de la vistule, Paris; Hippocrate-ECN, Faculté Dauphine, Place du Maréchal de Lattre de Tassigny, Paris; Conf-raphael, 10 rue Castex, Paris; Med XL / Conf +, 15 Rue Saint Bernard, Paris; Conférence Khalifa, Établissement d’enseignement supérieur libre, 134 Rue de Grenelle, Paris*) were collected respectively according to the data published publicly by the school of medicine, by the Official Journal of the French Republic and finally by the private training organizations on their respective websites. Data not publicly available was collected using a self-survey written in Word format (Microsoft, Redmond, USA) and delivered by email with the study’s letter of intent to inform the students concerned about the nature and purpose of the study, but also the confidential and anonymous treatment of all data collected, the prior declaration to the french national commission of informatics and liberties (CNIL) and finally the possibility left to everyone to opt out of the study. The survey consisted of 10 items (see Additional file 1) including a 3-items Likert-scale to measure the student motivation. Data collected in the framework of the study were in accordance to the French laws and subject to a declaration and approved by the CNIL (n°1907841v0).

### Statistical analysis

Descriptive analysis of quantitative variables was performed using means and standard deviations. Descriptive analysis of categorical variables was performed using enrollments and their percentages. In univariate analysis the quantitative variables were studied by the Pearson correlation coefficient and for the categorical variables by comparison of the mean by the Student or Kruskal-Wallis test according to the conditions of application. Statistically significant variables with a threshold of *p* <  0.05 in univariate analysis were used for multivariate analysis using a generalized linear regression whose results are expressed in β ± standard error (SE). Statistical analyzes were performed with SAS software version 9.4 (SAS Insitute, Inc., Cary, NC, USA) and the threshold of *p* <  0.05 was deemed significant.

## Results

### Descriptive results

**Fig. 1:**
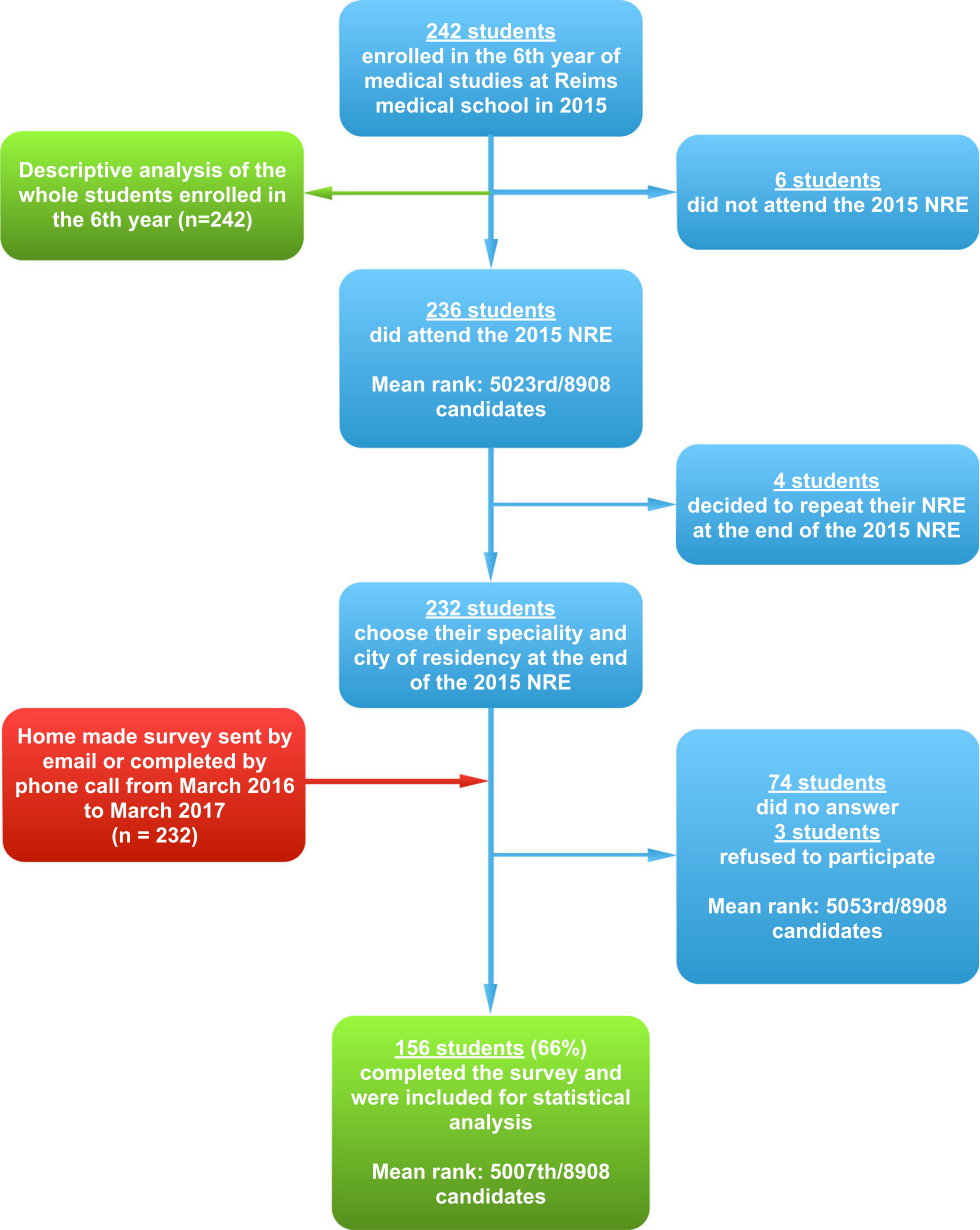
Flow chart

A total of 242 students were enrolled in the 6th year during the 2014–2015 academic year (Fig. 1). The mean age at the NRE was 25 ± 1.5 years (range: 22–34 years) (Tables 1, 2 and 3). This cohort was predominantly female (*n* = 141, 58%) for 101 male (42%). Out of these 242 students, 236 (98%) participated in the 2015 NRE (Fig. 1). The mean ranking of the Reims students at 2015 NRE was 5023rd/8908 candidates (Limits: 12th - 8743rd, SD: ± 2435 places). Ten students (4%) chose to repeat their 6th year at the end of the 2015 NRE in order to retake the examination in 2016, among them 4 students completed the 2015 NRE and were ranked (Fig. 1). The average of their rankings was 5859th / 8908 candidates. The remaining 6 students decided to repeat during the year and did not participate at the 2015 NRE and therefore were not ranked (Tables 1 and 2). Results of the univariate analysis were presented in Tables 1, 2 and 3.

**Table 1:** Description of the categorical variables of the cohort (*n* = 242) and univariate analysis of the predictive factors associated with a better ranking of the Reims students at the 2015 NRE

Variables	n (%)	Mean rank (± SD)	*p*
Population^1^	236 (98%)		
Gender			0.77
Female	138 (58%)	5062 (± 2261)	
Male	98 (42%)	4969 (± 2674)	
Answered survey			0.89
Yes	156 (66%)	5007 (± 2444)	
No	80 (34%)	5053 (± 2435)	
Decided to repeat the 2015 NRE in 2016			0.49
Yes	4 (2%)	5859 (± 1578)	
No	232 (98%)	5008 (± 2448)	
Repeating the NRE at 2015 NRE			0.74
Yes	23 (10%)	5181 (± 2554)	
No	213 (90%)	5006 (± 2428)	
Repeated the first year^a^			**< 0.0001**
Yes	89 (57%)	5743 (± 2026)	
No	68 (43%)	3953 (± 2610)	
Repetition during university course^2,a^			**< 0.0001**
Yes	39 (25%)	7020 (± 1705)	
No	117 (75%)	4337 (± 2283)	
Gaining a Master’s degree (first year)^a^			0.22
Yes	31 (20%)	4523 (± 2428)	
No	125 (80%)	5128 (± 2443)	
Gaining a Master’s degree (second year)^a^			0.44
Yes	3 (2%)	3930 (± 2648)	
No	153 (98%)	5029 (± 2445)	
Attendance at the hospital internship (4th year)^a^			
Yes	156 (100%)		
No	0 (0%)		
Attendance at the hospital internship (5th year)^a^			
Yes	156 (100%)		
No	0 (0%)		
Attendance at the hospital internship (6th year)^a,3^			**< 0.0001**
Yes	102 (65%)	5740 (± 2294)	
No	54 (35%)	3627 (± 2113)	
Attendance at the faculty lectures (1st year)^a^			0.74
Yes	148 (95%)	4980 (± 2481)	
No	7 (5%)	5300 (± 1641)	
Attendance at the faculty lectures (2nd year)^a^			0.71
Yes	91 (58%)	5070 (± 2389)	
No	65 (42%)	4921 (± 2535)	
Attendance at the faculty lectures (3rd year)^a^			0.87
Yes	77 (49%)	4974 (± 2353)	
No	79 (51%)	5041 (± 2544)	
Attendance at the faculty lectures (4th year)^a^			0.99
Yes	66 (42%)	5006 (± 2412)	
No	90 (58%)	5009 (± 2481)	
Attendance at the faculty lectures (5th year)^a^			0.45
Yes	58 (37%)	5006 (± 2412)	
No	98 (63%)	5009 (± 2481)	
Attendance at the faculty lectures (6th year)^a^			56
Yes	44 (28%)	5605 (± 2277)	
No	112 (72%)	4774 (± 2477)	
Intrinsic motivation^a^			
Highly motivated	74 (48%)	3664 (± 2112)	**< 0.0001**
Moderately motivated	61 (40%)	5634 (± 2016)	**< 0.0001**
Not motivated	21 (12%)	7870 (± 1078)	
Extrinsic motivation^4,a^			0.46
Yes	28 (18%)	4692 (± 2660)	
No	127 (82%)	5067 (± 2407)	
Targeted speciality^5,a^			0.62
Yes	129 (83%)	4964 (± 2403)	
No	27 (17%)	5220 (± 2667)	
Targeted city of residency^5,a^			**0.01**
Yes	82 (53%)	5465 (± 2444)	
No	74 (47%)	4501 (± 2358)	
Personal goal of ranking^5,a^	108 (46%)		

^1^All students who are completed the 2015 NRE. SD: standard-déviation. ^2^First and sixth years excluded. ^3^Motivation were in 100% of cases, revision and preparation of the NRE. ^4^Exstrinsic motivation that means the student feeling about their faculty supports in the NRE preparation and/or learning their future profession. ^5^Before completed the 2015 NRE. ^a^Unknown: *n* = 80 (33%); ^b^Unknown: *n* = 50 (21%); ^c^Unknown: *n* = 59 (25%)

boldface entries are statistically significant with a p < 0.05

**Table 2:** Description of categorical variables in the cohort (*n* = 242) and univariate analysis of predictive factors, relating to training conferences and national white NRE participation, associated with a better rank of the Reims students at 2015 NRE

Variables	n (%)	Mean rank (± SD)	*p*
Enrolled in free training conferences organized by the author			**0.04**
Yes	126 (53%)	4725 (± 2309)	
No	110 (47%)	5365 (± 2541)	
≥ 1 lecture			0.14
Yes	122 (52%)	4791 (± 2279)	
No	111 (48%)	5264 (± 2590)	
≥ 3 lectures			0.57
Yes	75 (33%)	4899 (± 2268)	
No	154 (67%)	5093 (± 2522)	
≥ 15 lectures			0.44
Yes	20 (9%)	4622 (± 2279)	
No	212 (91%)	5067 (± 2452)	
≥ 25 lectures			0.33
Yes	5 (2%)	6075 (± 1458)	
No	227 (98%)	5006 (± 2450)	
All the lectures (*n* = 30)			
Yes	0 (0%)		
No	236 (100%)		
Enrolled in paying training conferences organized by the Reims medical school (FERM^1^)			0.37
Yes	172 (73%)	4936 (± 2436)	
No	64 (27%)	5257 (± 2438)	
≥ 1 lecture			0.53
Yes	119 (77%)	4927 (± 2349)	
No	36 (23%)	5220 (± 2770)	
≥ 3 lectures			0.41
Yes	108 (70%)	4889 (± 2287)	
No	47 (30%)	5237 (± 2789)	
All the lectures			0.054
Yes	36 (23%)	4307 (± 2254)	
No	119 (77%)	5203 (± 2473)	
Paid private training conferences (4th year)^a^			
Yes	1 (0,6%)	4518	
No	155 (99,4%)	5011 (± 2452)	
Paid private training conferences (5th year)^a^			**0.03**
Yes	56 (36%)	4424 (± 2420)	
No	100 (64%)	5335 (± 2408)	
Paid private training conferences (6th year)^a^			0.17
Yes	109 (70%)	4830 (± 2332)	
No	47 (30%)	5419 (± 2667)	
*Hermès* conference^2^			**0.006**
Yes	63 (41%)	4389 (± 2263)	
No	92 (59%)	5472 (± 2461)	
*Hippocrate* conference^3^			0.26
Yes	2 (1%)	6946 (± 1833)	
No	153 (99%)	5006 (± 2436)	
*Conf +* conference^4^			0.75
Yes	30 (19%)	5157 (± 2226)	
No	125 (81%)	5001 (± 2489)	
*Khalifa* conference ^5^			0.74
Yes	6 (4%)	5363 (± 2345)	
No	149 (96%)	5018 (± 2444)	
*Raphael* conference ^6^			0.92
Yes	11 (7%)	5175 (± 2976)	
No	137 (93%)	5103 (± 2346)	
White NRE (4th year)^a^			0.61
Yes	2 (1%)	4125 (± 4045)	
No	154 (99%)	5019 (± 2436)	
White NRE (5th year)^a^			**0.007**
Yes	33 (21%)	3995 (± 2360)	
No	123 (89%)	5280 (± 2403)	
White NRE (6th year)^a^			**0.0001**
Yes	134 (86%)	4709 (± 2372)	
No	22 (14%)	6829 (± 2100)	
*Revue du praticien* November 2014^7,a^			**< 0.0001**
Yes	103 (67%)	4399 (± 2379)	
No	50 (33%)	6334 (± 2038)	
*Hippocrate* January 2015^3,b^			0.24
Yes	104 (56%)	4876 (± 2302)	
No	82 (44%)	5305 (± 2602)	
*Revue du praticien* March 2015^7,a^			**< 0.0001**
Yes	107 (69%)	4430 (± 2346)	
No	47 (31%)	6309 (± 2213)	
*Hippocrate* March 2015^3,c^			**0.004**
Yes	73 (41%)	4230 (± 2540)	
No	104 (59%)	5325 (± 2391)	

^1^Association of federation for the Reims medical students (FERM). ^2^*Hermès* conference, SASU, 11 rue de la vistule, Paris. ^3^*Hippocrate-ECN* conference, Faculté Dauphine, Place du Maréchal de Lattre de Tassigny, Paris. ^4^Med XL (*Conf +* conference), 15 Rue Saint Bernard, Paris. ^5^*Khalifa* conference, Établissement d’enseignement supérieur libre,134 Rue de Grenelle, Paris. ^6^*Conf-raphael* conference, 10 rue Castex, Paris. ^7^*ECN-blanches la Revue du Praticien* conference, Global Média Santé, 314, Bureau de la Colline, Saint-Cloud Cedex. ^a^Unknown: *n* = 80 (33%); ^b^Unknown: *n* = 50 (21%); ^c^Unknown: *n* = 59 (25%)

boldface entries are statistically significant with a p < 0.05

**Table 3:** Description of the quantitative variables of the population (*n* = 242) and univariate analysis of the predictive factors associated with a better ranking of the Reims students at the 2015 NRE

Variables	n	Mean rank (± SD)	*r*^d^	*p*
Mean age (year)	242	25.2 (± 1.5)	0.41	**< 0.0001**
Mean rank in first year^1^	207	122 (± 73)	0.29	**< 0.0001**
Mean results at the *CSCT*^2^ (in points)	230	573 (± 67)	−0.80	**< 0.0001**
Mean rank (SD) at the *CSCT*^2^	230	116 (± 67)	0.80	**< 0.0001**
White NRE (sixth year)^a^				
Median number of white NRE completed^a^	156	3 (range: 0–6)	− 0.44	**< 0.0001**
Mean results at Hippocrate white NRE January 2015^3,b^	97	449 (± 101)	−0.73	**< 0.0001**
Mean rank at Hippocrate white NRE January 2015^3,b^	97	2699 (± 1023)	0.79	**< 0.0001**
Mean results at Hippocrate white NRE March 2015^3,c^	63	557 (± 123)	−0.79	**< 0.0001**
Mean results at Hippocrate white NRE March 2015^3,c^	63	1812 (± 1053)	0.75	**< 0.0001**

^1^One student entered the second cycle of medical studies with a counter-equivalence and did not attend the first year ranking exam. SD: standard-deviation. ^2^*CSCT*: French certificate of clinical synthesis and therapeutics. ^3^*Hippocrate-ECN* conference, Faculté Dauphine, Place du Maréchal de Lattre de Tassigny, Paris. ^a^Unknown: *n* = 80 (33%); ^b^Unknown: *n* = 50 (21%); ^c^Unknown: *n* = 59 (25%). ^d^a negative correlation coefficient *r* was associated with a better ranking

boldface entries are statistically significant with a p < 0.05

### Results of the multivariate analysis

**Fig. 2:**
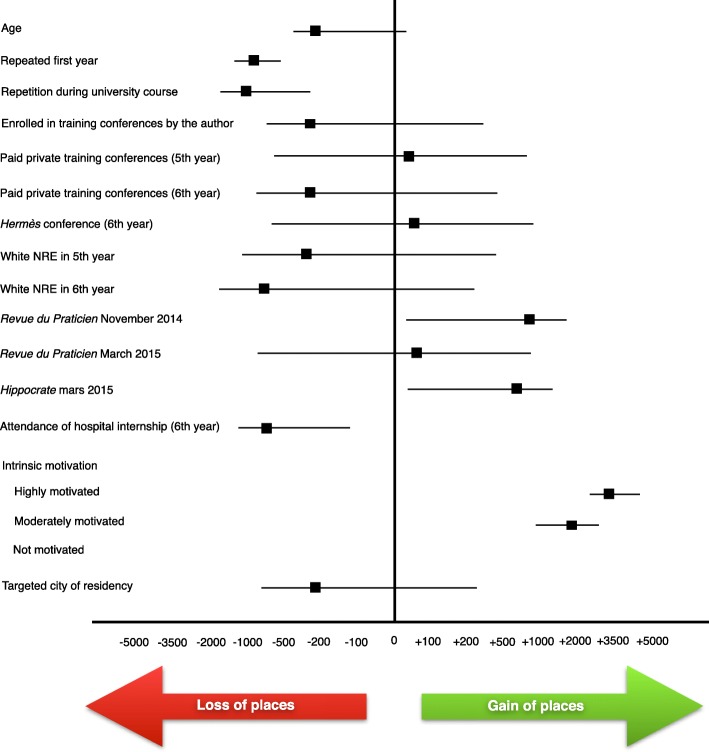
Forest plot of predictive factors associated with a better ranking of the Reims medical school students at 2015 NRE from the multivariate analysis

**Fig. 3:**
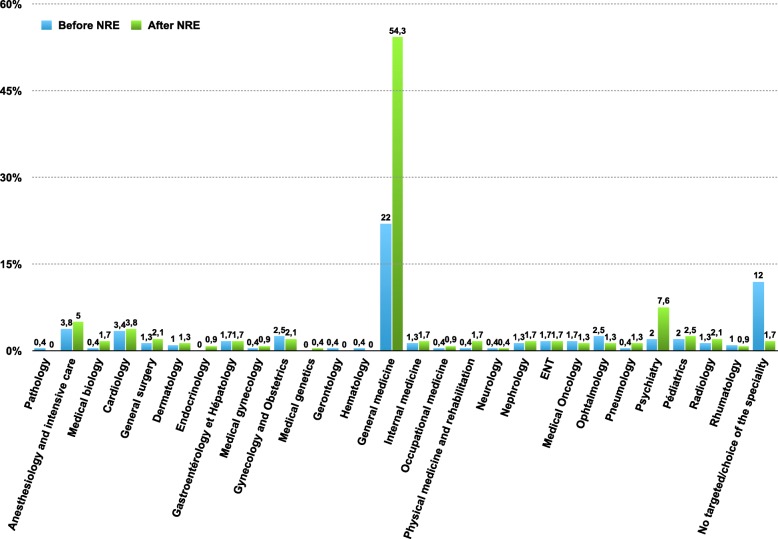
Targeted speciality before NRE and definitive choice after the NRE

**Fig. 4:**
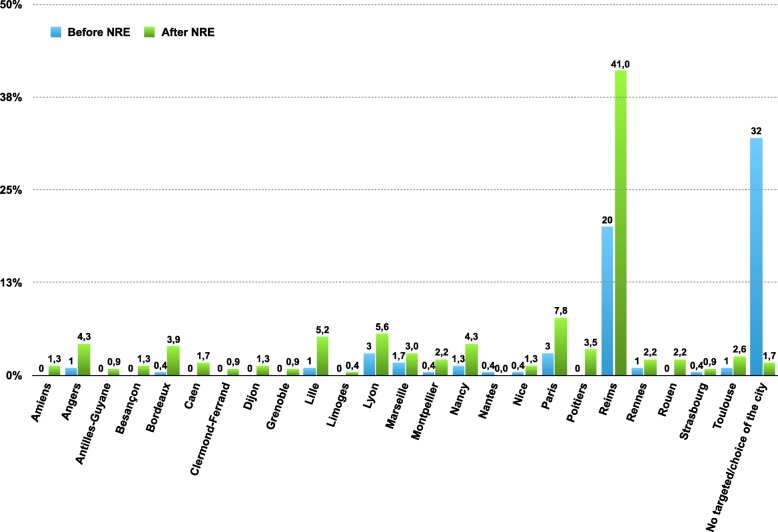
Targeted city of residency before NRE and definitive choice after the NRE

The factors independently associated with a better ranking at the NRE were the motivation for the preparation of the examination (gain of 3327 ± 527 places, *p* <  0.0001), to have participated in the NRE white test organized by *La Revue du Praticien* in November 2014 (gain of 869 ± 425 places, *p* <  0.04), to have participated in the NRE white examination organized by the *Hippocrate* conference in March 2015 (gain of 613 ± 297 places, p <  0.04). The factors independently associated with poor NRE ranking were repetition in their first year (loss of 1410 ± 286 places, p <  0.0001), repetition during their university course (loss of 1092 ± 385 places, *p* <  0.005), attendance of hospital internships in their 6th year (loss of 706 ± 298 places, *p* <  0.02) (Table 4 and Fig. 2). Student results, choice of specialty and city after the 2015 NRE are presented in Figs. 3 and 4.

**Table 4:** Multivariate analysis of the predictive factors associated with a better rank of the Reims students at 2015 NRE

Variables	β ± SE	IC 95%	*p*
Age	−225 ± 114 places	[−450; 0,28]	0.0503
Repeated the first year^a^	−1410 ± 286 places	[− 1976; − 844]	**< 0.0001**
Repetition during university course^1,a^	−1092 ± 385 places	[− 1853; − 331]	**0.005**
Enrolled in free training conferences organized by the author	− 281 ± 303 places	[− 880; 317]	0.35
Paid private training conferences (5th year)^a^	+ 44 ± 354 places	[−880; 317]	0.9
Paid private training conferences (6th year)^a^	− 249 ± 373 places	[− 987; 489]	0.5
*Hermès* conference^2^	+ 148 ± 348 places	[− 539; 836]	0.50
White NRE in 5th year^a^	− 371 ± 410 places	[− 1184; 442]	0.36
White NRE in 6th year^a^	− 850 ± 536 places	[− 1909; 210]	0.12
*Revue du Praticien* November 2014^3,a^	+ 869 ± 425 places	[27; 1711]	**0.04**
*Revue du Praticien* March 2015^3,a^	+ 61 ± 430 places	[− 790; 912]	0.88
*Hippocrate* mars 2015^4,c^	+ 613 ± 297 places	[26; 1200]	**0.04**
Attendance of hospital internship (6th year)^a^	−706 ± 298 places	[− 1297; − 116]	**0.02**
Intrinsic motivation^10,a^			
Highly motivated	+ 3327 ± 527 places	[2284; 4370]	**< 0.0001**
Moderately motivated	+ 1905 ± 474 places	[968; 2842]	**< 0.0001**
Not motivated	reference		
Targeted city of residency^12,a^	−255 ± 281 places	[− 810; 300]	0.36

^1^First and sixth years excluded. ^2^*Hermès* conference, SASU, 11 rue de la vistule, Paris. ^3^*ECN-blanches la Revue du Praticien* conference, Global Média Santé, 314, Bureau de la Colline, Saint-Cloud Cedex. ^4^*Hippocrate-ECN* conference, Faculté Dauphine, Place du Maréchal de Lattre de Tassigny, Paris. ^a^Unknown: *n* = 80 (33%); ^b^Unknown: *n* = 50 (21%); ^c^Unknown: *n* = 59 (25%)

boldface entries are statistically significant with a p < 0.05

## Discussion

The results of this study demonstrate for the first time that strong student motivation is independently and significantly associated with a gain of places at the NRE (average of 3327 places). While these results are in agreement with literature data in that a high level of intrinsic motivation often correlates with better exam success, none of the studies investigating predictive factors for NRE success previously published had shown this correlation [10–12]. Nevertheless, in 2015 Agrinier et al., showed that there was a tendency to gain places when intrinsic and extrinsic motivations was high, but their results were not confirmed in multivariate analysis [13]. Intrinsic motivation (defined as the driving force for the realization of an act from which the individual derives pleasure or satisfaction [14]) of the student was measured by a Likert-scale self-survey developed for this study. The students were overwhelmingly (88%) intrinsically motivated in their preparation for the NRE. This data seems in agreement with most previously published literature [11]. Indeed, Sobral showed in 2004 that the motivational profile of medical students was strongly self-determined and that the degree of intrinsic motivation was high [11]. However, the extrinsic motivation represented by faculty supports was low, only 18% of the surveyed students (66% of the 2015 cohort) felt supported or motivated by their faculty for NRE preparation but also for learning their future professional skills. Although this assessment by the student is, like the motivation, subjective, it is nevertheless very informative because 100% of the students questioned presented no difficulty for giving an answer and 100% of the answers were either affirmative or negative. The results of this study made from the 2015 class corroborate student feelings from previous and subsequent years in this medical school [15, 16]. The perception of failure by the medical students at this medical school is partly based on a non-controllable external factors such as, for example, the lack of teachers’ supervision, the examination conditions or difficulties making them resort to failure avoidance behaviours such as defensive pessimism, that is well known, depreciates intrinsic motivation [15, 17, 18, 19]. Nevertheless, despite a low extrinsic motivation represented mainly by the perception of a low motivational support on the part of the faculties, the students were mainly very motivated. However, it seems legitimate to assume that an increase in motivational support by faculties would reinforce the extrinsic motivation and inevitably increase the intrinsic self-motivation of the students, thus positively influencing their level of investment and their perseverance during NRE preparation. This could be an interesting and viable outcome strategy to improve the average rank of all students of this medical school at the national level.

This study also demonstrates for the second time, both independently and statistically, that the students NRE preparation by their participation in the national NRE white tests organized by private organizations is associated with an average gain of more than 600 places. Indeed, in 2010 Andujar et al., demonstrated that the participation in the NRE white tests was independently and significantly associated with a better rank at the NRE [12]. In 2015, Le Rouzic et al., demonstrated that participation in the NRE white test was significantly associated with a better ranking with a dose-effect relationship in the absence of adjustment for confounding factors [20]. While participation at the NRE white test has a positive impact on the NRE student ranking, attendance of the NRE training conferences, whether public or private, free or paid, was not independently and significantly associated with gain or loss of NRE places in multivariate analysis in this previous study [20]. Several hypotheses can be formulated to explain these results. Ninety-four percent of the students had attended a NRE training conferences during their 6th year, which severely limits the possibilities of comparison. Indeed, the vast majority of students want to give themselves every opportunity to succeed and therefore enroll in these NRE training conferences whether public or private. Nevertheless, it appears that students who have completed a NRE training conference in their 5th year have gained NRE places, but these significant results in univariate analysis have not been confirmed in the multivariate model. As well as, the students who followed the NRE training conference by *Hermès* (*Conference Hermès, SASU, 11 rue de la vistule, Paris*) in their 6th year had a significant gain in places compared to other students, but these results have not been confirmed in the multivariate model. A literature search seems contradictory about the impact of the NRE training conferences on the final NRE student rank. In 2011, Karila et al., did not demonstrate that regular attendance at medical school conferences was significantly and independently associated with a top-500 ranking at the ECN in multivariate analysis, although it appeared that this was the case in univariate analysis [10]. In 2015, Agrinier et al. reported that attendance of NRE training conferences was significantly and independently associated with an average gain of 873 NRE places with a dose-effect relationship of 63 places per conference attended [13]. More recently, in a study of predictive factors for success at the 2017 computerized-NRE and presented at the 76th congress of the French National Society of Internal Medicine (SNFMI), Bellamine et al., demonstrated that participation in training conferences organized by the medical school is significantly and independently associated with success at the computerized-NRE [21].

Our study also demonstrates for the first time that the 6th year hospital internships attendance was associated independently and significantly with an average loss of 706 NRE places. At the school of medicine of Reims, the hospital students (from the 4th to the 6th year) alternate 6 weeks periods of daily lectures (whole days) with university tests at the end of these last ones and 6 weeks periods of full-time hospital internships. Hospital internships are not being coordinated and correlated to the themes of the previous weeks of lectures. In the 6th year of medical studies, the hospital internships take place over 3 months with attendance only in the morning, the afternoon being reserved for lectures. Although mandatory and within the compulsory framework of the 6th year, the tolerance of the faculties-supervisors about the time of attendance in hospital internships for the students is not uniform across medical schools, or even within the same medical school [2]. Some have set up revision periods of varying durations (usually 1 month) by shifting the hospital internships after the NRE [2]. In this study, 35% of students surveyed (66% of the 2015 NRE) reported that they attended less than 50% of the time required during their 6th year clinical internship and were better ranked at the NRE. The reason for their absence was in 100% of cases for revision and preparation for the NRE. This result leads to several reflections, on the one hand because the acquisition of the whole second cycle program is truely daunting (345 items) and requires some “free” time wholely devoted to in depth learning. Indeed, it is illusory to believe that medicine could be learned entirely at the patient’s bedside or simply replace personal book work in favor of clinical internships. On the other hand, because the acquisition of the second cycle program is the minimum required for the exercise of quality medicine. The necessary exercise of consolidation of the six university years that it imposes represents a real test of intellectual endurance incompatible with a daily appearance on the hospital wards. In other words, the 6th year medical student probably does not take advantage of the bedside teaching in an optimal way and probably sacrifice precious revision time, and this study underlines that the organization of the working time of the students is critical to their success at NRE. In 2015, Gillois et al., demonstrated that integrated university-hospital training programs that coincide with faculty courses over time could provide a successful way for improving student success at NRE [22]. In addition, the results of the Agrinier et al. in 2015 seems to corroborate the results of our study [13].

Finally, this study confirms that the fact of having repeated the first year but also repeating during the university course (independently of the first year) was independently and significantly associated with an average loss of more than 1000 places at the NRE. Indeed, previous studies have also demonstrated this state of affairs [10, 12, 13, 22, 23].

The decree of the 20th July 2015 about the organization of the NRE leading to the third cycle of medical studies (residency) has transformed this examination since 2004 and it has now evolved into a computerized format called NREc (*ECNi*) set up in 2016. While the initial desire for such a reform was to overcome the NRE which was judged: 1/ Non-discriminatory due to the increase of the *numerus clausus* and the increasing number of medical students; 2/ Too cumbersome logistically, in manpower and cost; 3/ Of a limited educational value, favoring the excess of “cramming” focused on factual memorization [24], the first feedbacks do not seem to meet the objectives set [25] and the setbacks of the 2017 NREc events strongly questioned the value of these change [26]. Even though the reform of the medical residency competition with the implementation of the NRE in 2004 and the change of format that it implied did not change the profile of the well-ranked student [10, 23]. It is possible, it will be necessary to verify it, that the evolution of the NRE towards a computerized form, including also the change of testing format, will not affect the profile of the well-ranked student. Moreover, this seems to be confirmed by the recent work of Bellamine et al. [21]. Thus, it is clear that the profile of the well-ranked student in the medical residency competition before 2004, the NRE from 2004 to 2015 and the NREc from 2016 to today remains the same. While digitization and computing are great tools and they provide many educational services, they are still tools that serve the teacher and fortunately we cannot yet replace them.

This study has several limitations, the first one is related to its retrospective nature and the fact that students filled in a self-survey that could have a memory bias. The second one is that the response rate to the self-survey represents 66% of the 2015 NRE cohort of the medical school studied. Nevertheless, the study of the judgment criterion (NRE rank) of respondents to the survey against non-responders is almost identical, which makes it reasonable to assume that the sample surveyed seems representative of the entire student population. The third one is that the measurement of student motivation was performed by a 3-items Likert-scale. Taking into account the inherently subjective nature of this measure, it corresponds in practice to a decision that is easily expressed by students. This measure is certainly methodologically questionable, but nevertheless it has the advantage of being a very easy collected objective data by the students themselves. In addition, it could be interesting to explore the grit factor, or the perseverance and passion for long-term goals, which is a personality trait that is described as persevering through difficult tasks [27]. Indeed, recent reports showed that grit is a powerful predictor of medical student success [28–30]. Finally, this study considered only predictive factors for students themselves (exam success, attendance at lectures, attendance at hospital internships, participation in white NRE examination or hopeful selection of specialty, city, etc.). It would be useful objectively to determine the direct impact of the lectures (of their quality, their number or their intensity) on the rank of NRE, but such a study seems difficult to put in place. Nevertheless, many relevant indicators, albeit indirect ones, reflect the quality of medical school teaching, which seems to be the most important factor, consistent and everlasting in the success of the students at the NRE [3, 10, 31].

## Conclusions

The results of this study confirm that most medical students are highly motivated and invest in learning for their future profession. The external validity of this study shows that the profile of the “well-ranked” student is relatively stereotyped, reproducible and seems insensitive to the variables of time and place (medical school of origin). However, as suggested by the results of this study, all factors improving positively the student motivation in the preparation of the NRE should be highly considered as it was a huge prognostic factor of NRE success. In this way, considering to increase the number of white test examinations, decrease the time of attendance of hospital internship within the year of NRE associated to an improvement of extrinsic student motivation from faculty should be an interesting way to increase the intrinsic student motivation and the national rank of the medical school of Reims, but probably more broadly to all medical school. It would now be relevant to carry out other studies aimed at evaluating the impact of teaching methods and tools, but also the investment, motivation and profile of teachers from various medical schools to determine whether these factors influence the results of medical students at NRE and see if in the long-term they might allow to train more independent and competent young doctors.

## Supplementary information

**Additional file 1.** Survey items.

## Data Availability

The datasets that support the findings of this study are available from the corresponding author upon reasonable request.

## Abbreviations

FERM: Federation for Reims medical students association
NRE: National ranking examination
