# Supplementary material for: Predictive factors of success at the French National Ranking Examination (NRE): a retrospective study of the student performance from a French medical school
Source: BMC Med Educ. 2019 Dec 21;19:469. doi: 10.1186/s12909-019-1903-5 (PMC6925837; doi:10.1186/s12909-019-1903-5)
Supplement: Supplementary file 1 — Additional file 1. Survey items. [file 12909_2019_1903_MOESM1_ESM.docx]

Predictive factors of success at the French National Ranking Examination (NRE): a retrospective study of the student performance from the Medical School of Reims in 2015

**SURVEY ITEMS**

**1/** Repetition during the university course (first to sixth university year);

**2/** Gaining a Master’s degree (first year and second year) during their university education;

**3/** Participation in a public training conferences (organized by the medical school or residents) and / or organized by private organizations for NRE training (*Conférence Hermès, SASU, 11 rue de la vistule, Paris; Hippocrate-ECN, Faculté Dauphine, Place du Maréchal de Lattre de Tassigny, Paris; Conf-raphael, 10 rue Castex, Paris; Med XL / Conf +, 15 Rue Saint Bernard, Paris; Conférence Khalifa, Établissement d’enseignement supérieur libre, 134 Rue de Grenelle, Paris*);

**4/** Participation in white examinations organized nationally by private organizations (*ECN-blanches la Revue du Praticien, Global Média Santé, 314, Bureau de la Colline, Saint-Cloud Cedex; Hippocrate-ECN, Faculté Dauphine // Place du Maréchal de Lattre de Tassigny, Paris; Conf-raphael, 10 rue Castex, Paris; Conférence Hermès, SASU, 11 rue de la vistule, Paris*);

**5/** The attendance at their hospital internship (>50% of the required time) during the externship (fourth, fifth and sixth university year) and the reason for the absence (NRE preparation or personal reasons);

**6/** The faculty lectures attendance (>50% of the lectures) from the first to the sixth year and the reasons for the absence (preparation of the NRE or personal preference);

**7/** The degree of intrinsic motivation: not motivated or moderately motivated or highly motivated; **8/** Support and motivation (extrinsic motivation) from faculty in preparation for the NRE exam and/or learning their future profession;

**9/** The personal goal of ranking before completed the NRE;

**10/** The specialty and city targeted before passing the NRE.
